# The Impact of Preventive Health Behaviors and Risk Factors on Health Status of Ghanaians

**DOI:** 10.5539/gjhs.v5n5p124

**Published:** 2013-06-16

**Authors:** Bashiru I. I. Saeed, A. R. Abdul-Aziz, Samuel Blay Nguah, Xicang Zhao

**Affiliations:** 1 School of Finance and Economics, Jiangsu University, Zhenjiang, China; 2 Mathematics and Statistics Department, Kumasi Polytechnic, Kumasi, Ghana; 3 Paediatric Department, Komfo Anokye Teaching Hospital, Kumasi, Ghana

**Keywords:** preventive health behaviors, risk factors, SAGE study, health status, Ghana

## Abstract

The article here investigated the impact of Preventive Health Behaviors and Risk Factors as measures of Health Status of Ghanaians. We carry out a cross-sectional analysis of 5573 adults who participated and had indicated that they needed to state their health description in the three years prior to the phase 2007 World Health Organization, a study on Global Ageing and Adult health (SAGE) conducted in Ghana. The ordinal logistic regression model was employed for analysis using R. The results suggest that, there is incontrovertible evidence showing a strong relationship between preventive health behaviors and health status of Ghanaians. Again, the lifestyle of Ghanaians clearly manifests in their positive correlation with the good and moderate health state due to the high percentage (38.96% and 39.04%) respectively. The outcome points to a potential link with the Ghanaian social and health policies.

## 1. Introduction

Ghana is situated on the west coast of Africa, bordered by Burkina Faso to the north, and Côte D’Ivoire on the west and on the east by Togo. The country is divided into 10 administrative regions and Accra is its capital city with numerous ethnic groups and they speak more than 50 languages and dialects. Many preventable diseases such as malaria, trachoma, cholera and diarrhea in the country are sanitation and water related diseases. As one hygiene specialist, Mr Emmanuel T. Nyavor puts it, “poor sanitation poses greater health risk than HIV/AIDS” in the country (GNA, 2008). Some behavior risk factors clearly affect health in later life, as a variety of studies show. For instance, Østbye, Taylor and Jung (2002) examined the effects of various behaviors on a variety of health outcomes, including inability to work, self-reported health status, and hospitalization.

Meanwhile, factors that are associated with a wide range of health risk behaviors among adolescents in Brazil appear parallel to those found in industrialized countries: access to guns, substance use, and sexual abuse (Marcia, Helena, Marjorie, & Robert, 2001). Health-related behavior, defined as “a range of personal actions that influence health, disability, and mortality” (Umberson, Crosnoe, & Reczek, 2010), is one of the key mechanisms in the three downstream pathways to health in Berkman and colleagues’ model. Certain health behaviors, particularly exercise, eating well and adherence to medical regimens promote health and prevent illness. In contrast, smoking, excessive weight gain and substance abuse can compromise health (Umberson et al., 2010). Interestingly, Cacioppo et al. (2002) found no association between loneliness and obesity, smoking, alcohol use and physical activity among people. However, lonely and non-lonely adults differed in BMI and smoking, with lonely individuals more likely to engage in health-compromising behaviors (Lauder, Mummery, Jones & Caperchione, 2006). Data on participants in the Chicago Health, Aging, and Social Relations Study revealed that loneliness was associated with transitioning from physically active to sedentary status (Hawkley, Thisted, & Cacioppo, 2009). A recent study underscores that different health-related behaviors constitute separate and mostly unrelated factors among older adults (Cohen-Mansfield & Kivity, 2011).

Moreover, Harakeh, De Looze, Schrijvers, Van Dorsselaer and Volleberg (2012) identified unique and common predictors such as tobacco smoking, binge drinking, cannabis smoking, early sexual intercourse and so on as multiple health risk behaviors. However, other studies have suggested that preventive health behaviors may have an underlying basis and similar predictors. To date, most interventions targeting adolescent health risk behaviors have been behavior specific (Guilamo-Ramos, Litardo, & Jaccard, 2005). The importance of environmental factors such as the microsystem (e.g. Family, peers and school) and larger social contexts such as socioeconomic status and ethnic background have also been confirmed (Buhi & Goodson, 2007; Donovan, 2004; Galea, Nandi,& Vlahov, 2004). The relationships between the following variables have been studied: self-assessed health and personality (Goodwin & Engstrom, 2002); legal drug use, nutrition and sociodemographic characteristics (Kelleher, Friel, Gabhainn, & Tay, 2003; Zhu, Heo, Plankey, Faith & Allison, 2003); risky lifestyle behaviors (Schuit, Van Loon, Tijhuis, & Ocke, 2002; Roubenoff, Scrimshaw, Shetty, & Woo, 2000; Maeno et al., 2005); use of the health care system and life style (Tucker & Clegg, 2002); and isolated risky lifestyle behaviors such as smoking (Dedobbeleer, Beland, Contandriopoulos, & Adrian, 2004; Cho, Song, Smith, & Ebrahim, 2004). Yet behavioral risk factors cluster in individuals. For example, compared to nonsmokers, smokers have poorer diets, are less physically active, and consume more alcohol (Kranzler et al., 2002; Shah et al., 1993). Also, the prevention and management of many chronic illnesses require attention to multiple behavioral risk factors (Glasgow, Orleans, & Wagner, 2001). Analyses also suggest that a single overarching framework—the “5A’s”—can be used to guide screening and intervention efforts across a variety of behavioral risk factors, raising prospects for integrated approaches (Fiore et al., 2000; Whitlock, Orleans, Pender, & Allan, 2002).

Health improvement, the key objective of public health policy, requires solid factual information about the population's current health status (Griffiths, Jewell, & Donnelly, 2005). To make major improvements in the health status of Ghanaians and to reduce premature morbidity and mortality, preventive health behaviors and risk factors for diseases must be addressed. Accordingly, in this article, we sought to examine the impact of preventive health behaviors and risk factors such as tobacco usage, alcohol consumption, eating of fruits, eating of vegetables and involvement in moderate physical exercise on the health state of Ghanaians.

## 2. Material and Methods

### 2.1 Sampling Procedures

The data employed in this study were drawn from the World Health Organisation's Global Ageing and Adult Health (SAGE). This aims to address the gap in reliable data and scientific knowledge on ageing and health in low – and middle –income countries. SAGE is a longitudinal study with nationally representative samples of persons aged 50+ years in Ghana with a smaller sample of adults aged 18-49 years. Instruments are compatible with other large high-income country longitudinal ageing studies. Wave 1 was conducted during 2008-2009 and included a total of 4770 respondents aged 50+ and 803 aged 18-49.

Few low- and middle-income countries have data on levels and distribution of health and disability among the older population, much less on which morbidity trajectory their respective ageing populations are following: expansion of morbidity, where people are living longer with more disease and disability; compression of morbidity, where longevity increases but with delays in the age at onset and progression of disease; or a dynamic equilibrium, where longevity and disability rates both increase, but the severity of disability is not as severe. Large-scale longitudinal research in multiple settings is required to provide comparable information on health and well-being, as well as to track the impact of health intervention sand policies within and across countries. The World Health Organization (WHO) Study on global Ageing and adult health (SAGE) aims to address this gap with a global perspective. Its core consists of national longitudinal studies of older people in lower- and upper-middle-income brackets, complemented by research collaborations with existing local population-based research studies, comparisons with other domestic ageing studies, and also comparisons to studies outside Ghana. SAGE is a longitudinal study with nationally representative samples of persons aged 50+ years in Ghana, with comparison samples of younger adults aged 18–49 years in Ghana. The main aim is to generate valid, reliable and comparable information on a range of health and well-being outcomes of public health importance, in adult and older adult populations. The core SAGE countries provide a broad representation from different geographic regions of the world, different levels of economic development and different stages in the demographic and health transition, and include the two most populous countries of the world. SAGE is also designed to provide results that are comparable to ageing studies in high-income countries, such as the US Health and Retirement Study, the English Longitudinal Study on Ageing and the Collaborative Research on Ageing in Europe (COURAGE in Europe) Project in three countries. Data resource area and population coverage. Face-to-face interview was conducted in Ghana (2008–09). Multistage cluster sampling strategies were used where households were classified into one of two mutually exclusive categories:

(1) all persons aged 50 years and older were selected from households classified as ‘50+ households’; and
(2) one person aged 18–49 years was selected from a household classified as an ‘18–49 household’.

Household enumerations were carried out in the final sampling units. One household questionnaire was completed per household where a household informant and individual respondent need not be the same individual. One individual was selected from 18–49 households, whereas for 50+ households all individuals aged 50+ were invited to complete the individual interview. Proxy respondents were identified for selected individuals who were unable to complete the interview. Household-level analysis weights and person-level analysis weights were calculated for each country, which included sample selection and a post-stratification factor. Post stratification correction techniques used the most recent population estimates provided by the Ghana Statistical Service. The pooled Wave 1 national total for individual respondents included 4770 respondents aged 50+ and 803 aged 18–49.

A standardized survey instrument, set of methods, interviewer training and translation protocols are used in all SAGE countries. The SAGE household questionnaire consists of

(1) a household roster and modules about the dwelling, income, transfers in and out of the household, assets and expenditures;
(2) an individual questionnaire with modules on health and its determinants, disability, work history, risk factors, chronic conditions, care giving, subjective well-being, health care utilization and health system responsiveness;
(3) a proxy questionnaire about health, functioning, chronic conditions and health care utilization;
(4) a verbal autopsy module questionnaire to ascertain the probable cause of death for deaths in the household in the 24 months prior to interview or between interview waves; and
(5) appendices including Show cards to assist with the interviews. In addition, SAGE Wave 1 included anthropometric measurements (height, weight, waist and hip circumferences), blood pressure measures and a blood sample via finger prick, and performance tests including near and distant vision, a timed 4-m walk, grip strength, lung function and cognition.

International standards were used to harmonize education levels and occupations.

### 2.2 Model Specification

The primary variable of interest is:- the overall health state descriptions of Ghanaians. It is ordinal in nature with five outcomes namely; Very good, Good, Moderate, Bad and Very bad.

The appropriate choice of model for the analysis was, therefore, an ordinal logistic regression model (Maddala, 1983; McKelvery & Zavoina, 1975). The model is also an extension of the binary-outcome model (binary logit or probit model) as is in Greene (1990).

The model is a natural extension of the binary-outcome model and defined as (Greene, 1990):


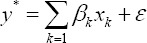


Where y* is a single choice in the ordered choices (very good, good, moderate, bad and very bad), ***β_κ_*** are estimators of the ordinal logit and it is assumed that follows a certain symmetric distribution with zero mean such as the normal or logistic distribution. The matrix ***Χ*** defines the ***preventive health behaviors and risk factors*** measured on each individual. Since the response outcome (health status) is categorical, the approach is to fit the ordinal logit model, each one comparing a given health state to ***very bad*** which is the referenced category. The choice of this referenced category is verified by the fact that ***very bad*** health state is of great concern in the Ghanaian society regardless of the tobacco usage and alcohol consumption.

Gives the general form for the probability that the observed (y=health status) falls into category (j=very good or good or moderate or bad or very bad), and the μs and the βs are to be estimated with an ordinal logit model.

### 2.3 The Ordinal Logit Model

For a response with five categories, the ordinal logit suggests that the effect of any explanatory variable would induce a change in the odds of responding to category ***very good*** instead of the rest, or ***very good*** or ***good*** or ***moderate*** or ***bad*** instead of ***very bad*** by a factor of exp(***β***). The software used in the analysis was R version 2.13.

## 3. Results

### 3.1 Data Description

Table 1 presents the descriptive statistics of the data used in the current study. It shows the total number and row percentage for characteristics of interest. After careful grouping of each factor into categories with meaningful sample sizes, additional model analyses were performed. The total number of observations for this study is 5573 distributed as shown. Most of the people who were asked to give their health state never use tobacco (75.6%). However, the same Ghanaians description of their health state was hugely associated with alcohol consumption (58.7%). The average fruits and vegetables eaten daily is two (2) for either. Interestingly, Ghanaians health state is found to be strongly associated with moderate physical exercise (69.3%). Ghanaians who responded they are in ***good*** and ***moderate*** health state are fairly equal, 38.96% and 39.04% respectively. The rest (***bad***, ***very good*** and ***very bad***) responded as low as 13.16%, 6.76% and 2.08% respectively.

**Table 1:** Descriptive statistics of the health state descriptions

Variable	Total	Percentage
**Health Status**		
*Very good*	345	6.8
*Good*	1989	39.0
*Moderate*	1993	39.0
*Bad*	672	13.2
*Very bad*	106	2.1
**Moderate Work**		
*No*	1558	31.0
*Yes*	3525	69.3
**Tobacco Use**		
*No*	3849	76.0
*Yes*	1241	24.4
**Alcohol Consumption**		
*No*	2103	41.3
*Yes*	2988	59.0
**Av. fruits eaten daily**	2	---
**Av. vegetables eaten daily**	2	---

### 3.2 Health State Model Specification and Estimation

To determine the significant ***preventive health behaviors and risk factors*** that influence ***health state descriptions*** of Ghanaians, an ordinal logit model was specified. Table 3 summarized the estimated odds ratio for the model. The parameters of the model were estimated using maximum likelihood approach. In Table 4 the estimates for each ***preventive health behaviors and risk factors*** are interpreted relative to the referenced category, ***very bad*.**

**Table 2:** General model diagnostic

Variables	(Mean) Std dev.	t value	P-value
Moderate Intensity Activity at Work		-7.7377	0.000
Ever used Tobacco?		0.3904	0.3
Ever consumed Alcohol?		0.5550	0.2
Av. fruits eaten daily	(2.2)1.89	-2.6877	0.003
Av. vegetables eaten daily	(2.0)0.94	0.7159	0.24

**Table 3:** Estimated odds ratio for health state model

Variables	Ordinal OR	Lower 95% C.I	Upper 95% C.I
**Moderate Work**			
*No*	Ref	---	---
*Yes*	0.639***,	0.57	0.716
**Tobacco Use**			
*No*	Ref	---	---
*Yes*	1.025	0.904	1.162
**Alcohol Consumption**			
*No*	Ref	---	---
*Yes*	1.031	0.925	1.15
**Av. fruits eaten daily**	0.957**	0.927	0.988
**Av. vegetables eaten daily**	1.021	0.964	1.082

Note: Av. and C.I, indicate Average and confidence interval respectively

In Table 3, the logit for servings of fruits eaten daily by an individual is about -0.044 and the corresponding effect on the odds after exponentiation is 0.957. Other things being equal, the odds of being classified as bad or moderate or good or very good versus very bad would be 0.957 times greater with a one-unit increase in the servings of fruits eaten daily.

Under the same circumstances, the odds of being classified as very good versus very bad would be 0.957 times greater with a one-unit increase in the servings of fruits eaten daily by an individual.

In a related development, the logit estimate for individuals whose work involved moderate physical exercise is approximately -0.448 and the corresponding effect on the odds after exponentiation is 0.639. This marginal effect suggests that the odds for those who were involved in moderate physical exercise during the work activity and classified as bad or moderate or good or very good instead of very bad are about 0.639 times as high as those whose work did not involve moderate physical exercise.

## 4. Discussion

In this cross-sectional study we sought to determine whether ***preventive health behaviors and risk factors have*** significant impact on ***health state descriptions*** of Ghanaians. It is evident from the specialist, Mr Emmanuel T. Nyavor puts it, “poor sanitation poses greater health risk than HIV/AIDS” in the country (GNA, 2008). Our study has also revealed that there is evidence that ***risk factorsresponses*** to ***health state descriptions*** are at variance with each other as corroborated by Umberson et al. (2010) who identified smoking, excessive weight gain and substance abuse can compromise health.

Also the nature of the data will not allow drawing conclusions about causal relationships. Again, the interviewee was to respond on his or her own health status. This is subjective, although, other researchers have proven that self-rated health status provides a valid prediction of the strong association between preventive health behaviors and risk factors. It has been established by Berner and Collins (1998) that some leading causes of mortality and morbidity among adolescents in both industrialized and developing nations are limited primarily to a relatively small number of preventable health risk behaviors that are often initiated in early adolescence. Many preventable diseases such as malaria, trachoma, cholera and diarrhea (the second single cause of child mortality in Ghana), in the country are sanitation and water related diseases

In another development, many studies point to the fact that ***risk factors*** contribute to the health status of the general population and continue to persist in old age. There is confirmation to that effect judging from a recent study conducted by Cohen-Mansfield & Kivitythat (2011) underscores the different health-related behaviors constituting separate and mostly unrelated factors among older adults. This study thus, supports the cross-sectional survey study by Harakeh et al. (2012) that examined unique and common predictors of tobacco smoking, binge drinking, cannabis smoking amongst others have an impact on health. Again, after controlling for ***preventive health behaviors***, a certain degree of imbalance remains. This is in agreement with Østbye et al. (2002) who looked longitudinally at the effects of various behaviors on a variety of health outcomes, including inability to work as they found in their study some behavior risk factors clearly affect health in later life.

Nevertheless our study has established enough evidence to the effect that response rate fails to capture everybody and this accounts for the shortfall.

## 5. Conclusion

In summary, there is incontrovertible evidence showing a strong relationship between preventive health behaviors and health status of Ghanaians. Again, the lifestyle of Ghanaians clearly manifests in their positive correlation with the ***good*** and ***moderate*** health state due to the high percentage (38.96% and 39.04%) respectively. The outcome points to a potential link with the Ghanaian social and health policies. Nevertheless, the preventive health behaviors and risk factors could be structured to take into account explicitly the underlying assumptions involved in health state descriptions. This could help health practitioners concerned with health care delivery to appreciate the problems and advise the Ministry of Health accordingly.
